# Production of Biopharmaceuticals in *Nicotiana benthamiana*—Axillary Stem Growth as a Key Determinant of Total Protein Yield

**DOI:** 10.3389/fpls.2019.00735

**Published:** 2019-06-11

**Authors:** Marie-Claire Goulet, Linda Gaudreau, Marielle Gagné, Anne-Marie Maltais, Ann-Catherine Laliberté, Gilbert Éthier, Nicole Bechtold, Michèle Martel, Marc-André D’Aoust, André Gosselin, Steeve Pepin, Dominique Michaud

**Affiliations:** ^1^Centre de recherche et d’innovation sur les végétaux, Faculté des Sciences de l’agriculture et de l’alimentation, Université Laval, Québec, QC, Canada; ^2^Medicago Inc., Québec, QC, Canada

**Keywords:** plant molecular farming, *Nicotiana benthamiana*, influenza virus hemagglutinin H1, light regime, apical pruning, hormone treatment, 6-benzylaminopurine

## Abstract

Data are scarce about the influence of basic cultural conditions on growth patterns and overall performance of plants used as heterologous production hosts for protein pharmaceuticals. Higher plants are complex organisms with young, mature, and senescing organs that show distinct metabolic backgrounds and differ in their ability to sustain foreign protein expression and accumulation. Here, we used the transient protein expression host *Nicotiana benthamiana* as a model to map the accumulation profile of influenza virus hemagglutinin H1, a clinically promising vaccine antigen, at the whole plant scale. Greenhouse-grown plants submitted to different light regimes, submitted to apical bud pruning, or treated with the axillary growth-promoting cytokinin 6-benzylaminopurine were vacuum-infiltrated with agrobacteria harboring a DNA sequence for H1 and allowed to express the viral antigen for 7 days in growth chamber under similar environmental conditions. Our data highlight the importance of young leaves on H1 yield per plant, unlike older leaves which account for a significant part of the plant biomass but contribute little to total antigen titer. Our data also highlight the key contribution of axillary stem leaves, which contribute more than 50% of total yield under certain conditions despite representing only one-third of the total biomass. These findings underline the relevance of both considering main stem leaves and axillary stem leaves while modeling heterologous protein production in *N. benthamiana.* They also demonstrate the potential of exogenously applied growth-promoting hormones to modulate host plant architecture for improvement of protein yields.

## Introduction

Several plants are used as heterologous expression hosts to produce recombinant proteins of medical interest, notably including the wild relative of tobacco *Nicotiana benthamiana* (Bally et al., 2018). This small plant from Australia presents a number of traits, such as a fast growth rate and a natural ability to express heterologous gene sequences, that make it particularly well suited to the production of biopharmaceuticals (Lomonossoff and D’Aoust, 2016). Efficient procedures have been devised for the transient expression of recombinant proteins in *N. benthamiana* that often involve the vacuum infiltration of leaf tissue with agrobacteria harboring a DNA transgene for the protein of interest delivered by either a viral replicon or a binary vector system (Leuzinger et al., 2013; Norkunas et al., 2018). A variety of therapeutic and diagnostic proteins have been produced in agroinfiltrated *N. benthamiana* plants recently, including mammalian antibodies (Qiu et al., 2014; Jutras et al., 2016; Li et al., 2016; Lee et al., 2018; Marusic et al., 2018; Kommineni et al., 2019; Kopertekh et al., 2019), viral antigens (Jutras et al., 2015; Tusé et al., 2015; Regnard et al., 2017; Mbewana et al., 2018; Roychowdhury et al., 2018; Tottey et al., 2018; Vanmarsenille et al., 2018; Zhumabek et al., 2018; Laughlin et al., 2019), and other proteins of potential clinical value (Rattanapisit et al., 2017, 2019; Fu et al., 2018; Ramirez-Alanis et al., 2018; Silberstein et al., 2018).

In practice, recombinant protein yield in plant (e.g., *N. benthamiana*) transient expression settings depends on the net amount of protein per gram of leaf tissue; the leaf biomass per plant prior to agroinfiltration; and the number of plants per growing area before plant tissue downstream processing (Fujiuchi et al., 2016; Shang et al., 2018). Several studies have reported the development of molecular tools or strategies to improve recombinant protein yield and quality in *N. benthamiana* leaves, notably to maximize the structural resemblance between plant-made proteins and their original counterparts or to protect those proteins that show limited stability in plant cell environments (Faye et al., 2005; Gomord et al., 2010). Recent studies have for instance described the expression of an accessory oligosaccharyltransferase to maximize *N*-glycan occupancy on maturing glycoproteins (Castilho et al., 2018), the *in situ* modulation of glycan-processing enzymes to optimize protein glycosylation patterns (Kallolimath et al., 2016; Li et al., 2016; Jansing et al., 2018), or the expression of protease inhibitors to prevent unintended hydrolysis by resident proteases (Goulet et al., 2012; Robert et al., 2016; Grosse-Holz et al., 2018; Jutras et al., 2019). Other studies have described the expression of a viral proton channel to stabilize labile proteins in the cell secretory pathway (Jutras et al., 2015, 2018), the expression of a human convertase to promote the post-translational proteolytic processing of clinically useful proteins *in vivo* (Wilbers et al., 2016; Mamedov et al., 2019), or the exogenous induction of the jasmonic acid defense pathway to reduce endogenous protein content in leaf tissue prior to recombinant protein purification (Robert et al., 2015). In parallel, studies have documented the effects of cultural practices on *N. benthamiana* growth and leaf biomass production before agroinfiltration (Fujiuchi et al., 2014; Shang et al., 2018), the influence of environmental parameters in growth chambers following agroinfiltration (Matsuda et al., 2017, 2018), or the impact of plant density on overall protein yield in specific culture settings (Fujiuchi et al., 2017; Shang et al., 2018).

Our goal in this study was to document eventual relationships between cultural practices, host plant growth pattern and recombinant protein yield in *N. benthamiana* leaves. Higher plants are complex organisms with young, mature, and senescing organs that show distinct metabolic backgrounds and differ in their ability to sustain protein biosynthesis and accumulation. In particular, low protein content in aging leaves due to reduced synthesis and increased degradation for nitrogen recycling toward growing organs has a strong impact on soluble protein distribution in the plant (Avila-Ospina et al., 2014; Havé et al., 2017). Accordingly, mammalian antibody yields in transgenic or agroinfiltrated tobacco plants were found to be low in old (bottom) leaves compared to younger leaves (Stevens et al., 2000; Buyel and Fischer, 2012). Likewise, antibody accumulation patterns in agroinfiltrated *N. benthamiana* leaves are age-dependent and closely match the distribution pattern of endogenous proteins in young and older leaves of the main stem (Robert et al., 2013; Jutras et al., 2016). A question at this stage is whether commonly adopted cultural practices in greenhouse settings may influence the overall yield of a recombinant protein *via* measurable effects on the host plant leaf pattern. A related question is whether such eventual effects of cultural practices can be harnessed to generate protein yield gains on a whole plant basis. We here addressed these questions using supplemental lighting, apical bud pruning, and axillary growth-promoting hormone treatments as model cultural practices eventually impacting host plant growth. *N. benthamiana* plants agroinfiltrated to express the vaccine antigen influenza virus A hemagglutinin H1 were used as a model protein factory of economic relevance (D’Aoust et al., 2010).

## Materials and Methods

### Plant Growth Conditions

Experiments took place at Laval University in Québec City, QC, Canada (46°46′ N, 71°16′ W). *N. benthamiana* plants were grown from seeds kindly provided by Medicago Inc. (Québec City, QC). The seeds were soaked in water for 24 h at 20°C and then placed in peat moss substrate for germination in a PGR15 growth chamber (Conviron, Winnipeg MB, Canada). Seedlings were selected after 2 weeks based on uniformity, transplanted in 350-ml plastic pots filled with peat moss substrate, and let to grow in greenhouse for an additional 3 weeks at a culture density of 33 plants·m^−2^ under different cultural conditions before leaf agroinfiltration (see below). Day and night temperatures in the greenhouse were maintained at 29 and 27°C, respectively. Supplemental lighting was provided by 400-W high-pressure sodium lamps installed above the plant canopy (P.L. Light Systems, Beamsville ON, Canada). The plants were irrigated as needed with the Plant-Prod 12-2-14 Optimum complete nutrient solution supplemented with the Plant-Prod Chelated Micronutrient Mix (Plant Products, Laval QC, Canada). Electrical conductivity in the nutrient solution was maintained at 1.6 dS·m^−1^ for 1 week after transplantation, and then increased at 2.6 and 3.6 dS·m^−1^ for the second and third weeks, respectively.

### Light Regime Treatments

Supplemental lighting trials for biomass production involved two photosynthetic photon flux densities (PPFDs) and two photoperiods, for a total of four treatments. Treatment 1 involved a 16-h day/8-h night photoperiod with a PPFD of 80 μmol/m^2^·s; Treatment 2, a 16-h day/8-h night photoperiod with a PPFD of 160 μmol/m^2^·s; Treatment 3, a 24-h day photoperiod with a PPFD with 80 μmol/m^2^·s; and Treatment 4, a 24-h day photoperiod with a PPFD of 160 μmol/m^2^·s. The plants were left to grow in greenhouse for 3 weeks under either light regime before use for leaf agroinfiltration. Light integrals for the 3-week growth period were estimated at ~200, 275, 240, and 365 mol·m^−2^ for Treatments 1, 2, 3, and 4, respectively.

### Pruning Treatments

Pruning treatments to promote axillary stem (or “secondary stem”) growth involved removal of the main stem (“primary stem”) apical bud 5, 7, or 12 days after seedling transplantation in plastic pots. The plants were then left to grow for an additional 16, 14, or 9 days in greenhouse under the same conditions, for a total growth period of 3 weeks before leaf agroinfiltration. Light conditions for this trial were as above for Treatment 4.

### Hormone Treatments

Growth hormone treatments to promote axillary stem growth involved exogenous application of the synthetic cytokinin 6-benzylaminopurine (6-BAP) (Bio Basic, Markham ON, Canada). Seedling transplants left to grow in greenhouse were sprayed 7 or 12 days after transplantation with the cytokinin diluted in water at working doses of 100, 500, or 1,000 ppm. The treated plants were kept in greenhouse for an additional 13 or 8 days, respectively, for a total growth period of 20 days before leaf agroinfiltration. Light conditions for this trial were as above for Treatment 4.

### Leaf Agroinfiltration and H1 Heterologous Expression

Three plants from each treatment were collected randomly at the end of the growth period to determine the leaf fresh weight (LFW) of primary and secondary stem leaves at agroinfiltration. Five other plants were collected for vacuum infiltration at Laval U (Jutras et al., 2018) or at Medicago research facility (Québec City QC) with agrobacteria harboring a Medicago proprietary vector with a DNA coding sequence for the H1 antigen of influenza virus, strain A/California/07/09 driven by the Cauliflower mosaic virus 35S constitutive promoter (Jutras et al., 2015). Agroinfiltrated plants were incubated in PGR15 growth chambers (Conviron) for 7 days under ambient CO_2_ concentration. Conditions in the growth chambers were as follows: air temperature set at 20°C, a relative humidity of 70%, a plant culture density of 55 plants·m^−2^, tap water irrigation provided as needed, and a PPFD of 150 μmol·m^−2^·s^−1^ provided 16 h a day by fluorescent tubes installed above and within the plant canopy. Primary and secondary stem leaves were harvested separately from each plant 7 days after agroinfiltration, weighed to measure LFW at harvest, and frozen at −80°C until use for H1 antigen determinations.

### H1 Antigen Hemagglutination Assay

Leaf tissue for H1 activity determination was broken with 2.8-mm zirconium ceramic oxide beads in an OmniBead Ruptor 24 homogenizer (Omni International, Kennesaw GA, USA). Leaf proteins were extracted in two volumes (i.e., 1.5 g leaf tissue per 3 ml buffer) of ice-cold 50 mM Tris–HCl, pH 8.0, containing 500 mM NaCl, 1 mM phenylmethylsulfonyl fluoride, and 2 mM sodium metabisulfite. The leaf homogenate was centrifuged at 4°C for 10 min at 20,000× *g* to recover soluble proteins in the supernatant. Protein content was determined according to Bradford (1976), with bovine serum albumin (Sigma-Aldrich) as a protein standard. H1 hemagglutination activity per LFW unit was determined using a Medicago-standardized hemagglutination assay (D’Aoust et al., 2008) based on the method of Nayak and Reichl (2004). Serial double dilutions of the test samples (100 μl) were made in V-bottomed 96-well microtiter plates containing 100 μl of phosphate-buffered saline, to leave 100 μl of diluted sample per well. One hundred μl of a 0.25% (w/v) Turkey red blood cell suspension (Bio Link Inc., Syracuse NY, USA) was added to each well and the plates were incubated for 2 h at 20°C. Reciprocal of the highest dilution showing complete hemagglutination was recorded as HA activity. H1 yield per plant (or per “leaf production unit”; see below) was calculated by multiplying HA activity per LFW by the LFW per plant (or per leaf production unit) at harvest.

### Experimental Designs and Statistical Analyses

Lighting trials followed a split-plot experimental design with seven replications in time (each including two sub-replicates) with at least 15 plants per replication unit, two photoperiods (16 vs 24 h) as main plots and two PPFDs (80 vs 160 μmol/m^2^·s) as sub-plots, for a total of four treatments. The pruning trial, with four treatments, followed a factorial experimental design with two replications per trial repeated two times, and five plants per replication unit. The growth hormone trial, with four treatments, followed a factorial experimental design with three replications and five plants per replication unit. Data in each trial were processed by an analysis of variance (ANOVA), using the GLIMMIX procedure of SAS, v. 9.4 (SAS Institute Inc., Cary NC, USA). Protected Fisher LSD (lighting trial) or Tukey’s (pruning/hormone trials) multiple comparison tests were used for mean separation following statistically significant ANOVA’s, using an alpha value threshold of 5%.

## Results and Discussion

**Figure 1:**
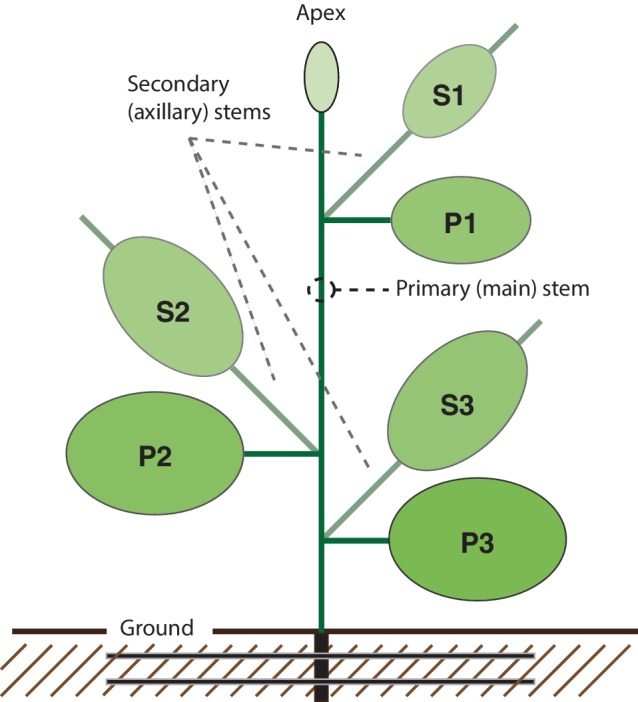
Schematic overview of the *N. benthamiana* protein factory. The plant is composed of six “leaf production units,” each regrouping leaves of similar physiological age. P units regroup top (young) leaves (P1), middle (mature) leaves (P2), and bottom (older) leaves (P3) of the main (primary) stem. S units regroup axillary (secondary) stems and leaves emerged from P1 leaves (S1), P2 leaves (S2), and P3 leaves (S3). Main stem apex (Apex) includes the main stem apical bud and small leaves appeared after agroinfiltration during the protein expression period.

The main goal of this study was to assess the effects of common cultural practices such as supplemental lighting, apical pruning, or growth hormone treatment on the overall yield of a clinically useful vaccine antigen transiently expressed in *N. benthamiana*. Given the well-known effects of cultural practices on host plant development patterns, a first step toward this goal was to evaluate the contribution of primary and secondary stem leaves to total H1 yield per plant, also considering leaf position relative to the primary stem apex given the link previously reported between leaf age and recombinant protein production in *N. benthamiana* (Robert et al., 2013). To this end, we divided the plant into six parts, or leaf production unit, each regrouping leaves of comparable physiological age (Figure 1). Primary (P) and secondary (S) stem leaves were considered separately to determine the relative contribution of axillary growth to total protein yield. Both groups of leaves were subdivided into three categories (1, 2, and 3) corresponding to, or emerged from, top (young) (P1, S1), middle (mature) (P2, S2), or bottom (oldest) (P3, S3) leaves down from the main stem apex, to take the impact of leaf aging into account.

### The H1 Antigen Is Not Distributed Evenly in the Plant

**Figure 2:**
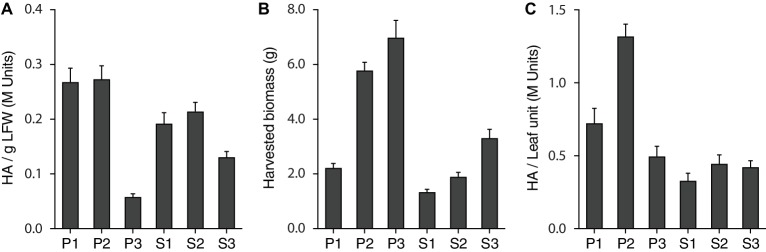
The H1 antigen is not evenly distributed in the plant. H1 hemagglutination activity **(A)**, harvested leaf biomass following H1 expression **(B)**, and total H1 yield **(C)** are here given for the six leaf production units (P1–P3, S1–S3). Data correspond to mean consolidated values calculated with the whole set of data produced for Treatments 1–4 during the PPFD/photoperiod lighting trial. Each bar is the mean of 56 independent (replication) values ± se.

We first produced a reference table for the relative contribution of each leaf production unit to total biomass and H1 yield on a whole plant basis (Table 1, Figure 2). Average, consolidated values were calculated for harvested biomass and H1 activity per g LFW using the whole set of data produced for Treatments 1–4 during the lighting trial. In line with Robert et al. (2013) reporting high-yield expression of a recombinant mammalian antibody in young leaves of the main stem, P1 and P2 were by far the most productive units for H1, both showing a specific hemagglutinin activity greater than 0.25 M Units/g LFW (Figure 2A) and accounting, together, for 56% of total H1 activity units in the plant for less than 40% of the leaf biomass (Table 1). In sharp contrast, P3 showed a specific H1 activity lower than 0.05 M Units/g LFW and accounted for only 12% of total H1 activity despite representing almost one-third of the leaf biomass. As for P units, a leaf position-related decline of H1 activity was observed in the S units on a LFW basis (Figure 2A), associated with an H1 yield/leaf biomass contribution ratio lower than 1 for S3 compared to a ratio well above this value for the S1 and S2 units (Table 1). As expected, given the less advanced physiological age of S3 leaves compared to P3 leaves from which they emerged, a specific H1 activity of ~0.12 M Units/g LFW was measured for the S3 unit, more than twice the activity measured for P3 (Figure 2A). This, along with a harvested biomass for S3 much greater than the biomass harvested for S1 and S2, prevented the onset of a leaf age-related decline of total H1 yield in S production units such as that observed in the P units (Figures 2B,C).

**Table 1:** Relative contribution of P and S leaf production units to total biomass and H1 yield per plant following heterologous expression.^¥^

Leaf unit	Contribution to yield per plant (%)	Contribution to leaf biomass (%)	Yield/biomass contribution ratio
P1	20.1	10.6	1.9
P2	35.8	27.5	1.3
P3	11.7	32.3	0.4
S1	9.1	6.1	1.5
S2	11.9	8.5	1.4
S3	11.5	14.9	0.8
P	67.6	70.5	1.0
S	32.4	29.5	1.1

¥ Data are expressed relative to total leaf biomass or H1 antigen yield per plant (100%) after heterologous expression. Each value is the mean of 56 independent (replication) values.

### Axillary Stem Leaves Act As H1 Yield Contributors and Source Organs for Young, High-Protein Expressing Leaves on the Main Stem

We conducted a complementary experiment to determine whether P3 leaves, although producing a small amount of H1 given their large biomass, would contribute to the overall H1 yield per plant by behaving as source organs for younger leaves actively expressing the recombinant antigen (Table 2). The possible contribution of axillary leaves as source organs was assessed in parallel to underscore an eventual reliance of young, fast-growing P1 and P2 leaves on external metabolic resources from the S production units during H1 expression. Seedling transplants were left to grow for 3 weeks in greenhouse and leaf-infiltrated with agrobacteria harboring the H1 gene construct. Right after infiltration, the oldest six leaves on the main stem (corresponding to P3), all axillary stems (bearing S unit leaves), or both groups of leaves were gently removed, keeping intact the main stem, the P1/P2 units, the P3 unit, and/or the S units before H1 expression in growth chamber. Despite a total leaf biomass reduced by 28%, P3-excised plants produced as much H1 as non-excised control plants under the same cultural conditions (Table 2). As expected, given the significant contribution of S units to total H1 yield per plant, axillary stem-excised plants showed a total H1 yield loss of 22% for a leaf biomass reduced by 20%, in close match with the yield/biomass contribution ratio of 1.1 calculated above for the S units (see Table 1). By comparison, plants devoid of both the S and P3 leaves showed a total yield loss of 38% for a total biomass reduced by 47%, about two times a consolidated yield loss of 20% calculated for the S and P3 units excised separately. These data confirmed the reliance of P1 and P2 production units on metabolic resources provided by the S and P3 units during H1 expression. They also underlined the key role of axillary stem leaves in *N. benthamiana* used as a protein factory, both as contributors to recombinant protein yield and as source organs to sustain the yield contribution of young, highly-expressing leaf production units on the main stem.

**Table 2:** Impact of P3 and/or S1–S3 units removal at infiltration on total biomass and H1 yield per plant following heterologous expression.^¥^

Production units removed	Biomass loss (%)	Yield loss (%)
P3	27.7 ± 0.05	−2.1 ± 0.08
S1, S2, S3	20.2 ± 0.03	22.0 ± 0.12
P3, S1, S2, S3	47.3 ± 0.04	38.4 ± 0.07

¥ Data are expressed relative to non-excised control plants (0% loss). Each value is the mean of three independent (replication) values ± se.

### High PPFD and an Extended Photoperiod Enhance Total H1 Yield Per Plant *via* a Positive Effect on Axillary Stem Growth

**Figure 3:**
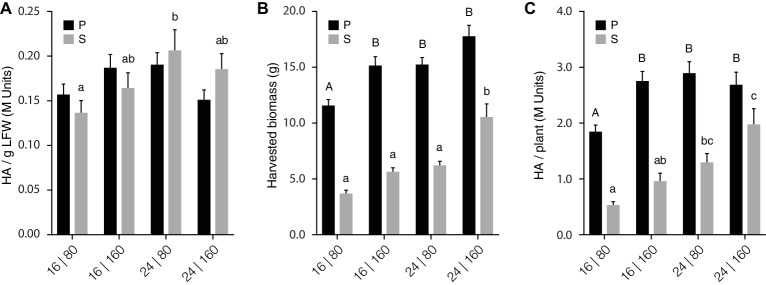
PPFD and light photoperiod influence total H1 yield *via* their strong impact on axillary growth. H1 hemagglutination activity **(A)**, leaf biomass following H1 expression, **(B)** and total H1 yield **(C)** for each leaf production unit are given for the four PPFD/photoperiod treatments. Each bar is the mean of 14 independent (replication) values ± se. On each panel, bars with different letters (uppercase letters for P units, lowercase letters for S units) indicate statistically significant differences between treatments (post-ANOVA Tukey’s test; *p* < 0.05). P, leaf production units P1–P3; S, leaf production units S1–S3.

We took a closer look at our lighting trial data to characterize the impact of light regime on leaf biomass production and H1 yield per leaf production unit (Figure 3, Supplementary Table S1). Light intensity and day length directly determine the amount of light available to plants for photosynthesis and supplemental lighting generally has a positive impact on biomass production in greenhouse settings (Dorais and Gosselin, 2002). Accordingly, the overall yield of mouse antibody MGR48 in transgenic tobacco plants was promoted under high-light conditions, mostly *via* a positive effect on leaf biomass production (Stevens et al., 2000). Similarly, increasing PPFD from 80 to 160 μmol/m^2^·s here had a limited impact on H1 activity per LFW in both P and S leaves (Figure 3A) but a strong positive impact on leaf biomass and total H1 yield in both groups of leaves (Figures 3B,C). For instance, leaf biomass and total H1 yield were improved by 25–50% in P leaves provided 16 h/day with the high PPFD regime of 160 μmol/m^2^·s (Treatment 2; see Light regime treatments, above). Likewise, leaf biomass and total H1 yield were improved by more than 60% in S leaves provided 24 h/day with the highest PPFD (Treatment 4). Extending daylight photoperiod from 16 to 24 h also had a positive impact on leaf biomass and H1 yield per plant, for instance improving H1 yield by ~50% in P units at low PPFD (Treatment 3) or by ~100% in S units at high PPFD (Treatment 4) (Figure 3C).

A high PPFD, or an extended photoperiod, were sufficient to reach maximal biomass or H1 yield gains for the P units under our experimental conditions (Figures 3B,C). For instance, comparable biomass or H1 yield gains were obtained for P leaves provided 16 h/day with the highest PPFD (Treatment 2), 24 h/day with the lowest PPFD (Treatment 3), or 24 h/day with the highest PPFD (Treatment 4). Likewise, a total H1 yield/biomass contribution ratio of 0.9 was calculated for P leaves grown under Treatment 4, compared to a ratio of 1.0 for plants grown under the other three light regimes (Supplementary Table S1). By comparison, additive effects of PPFD and day photoperiod were observed for the S units of plants grown under Treatment 4 (Figures 3B,C). For instance, leaf biomass and H1 yield per plant were higher in S leaves under this treatment than in S leaves grown under Treatment 2 or Treatment 3. Similarly, the H1 yield/biomass ratio in S leaves increased with light intensity or day photoperiod, from 0.9 for Treatment 1 to 1.0 or 1.1 for Treatments 2 or 4, respectively (Supplementary Table S1). These data pointed overall to the limited potential of P units for additional yield gains under commonly used lighting regimes in greenhouse settings. By contrast, they suggested the potential of axillary growth-promoting cultural practices for additional protein yield gains on a whole plant basis.

### Tip Pruning and 6-BAP Treatment Both Promote Axillary Stem Growth But Differentially Impact Total H1 Yield Per Plant

**Figure 4:**
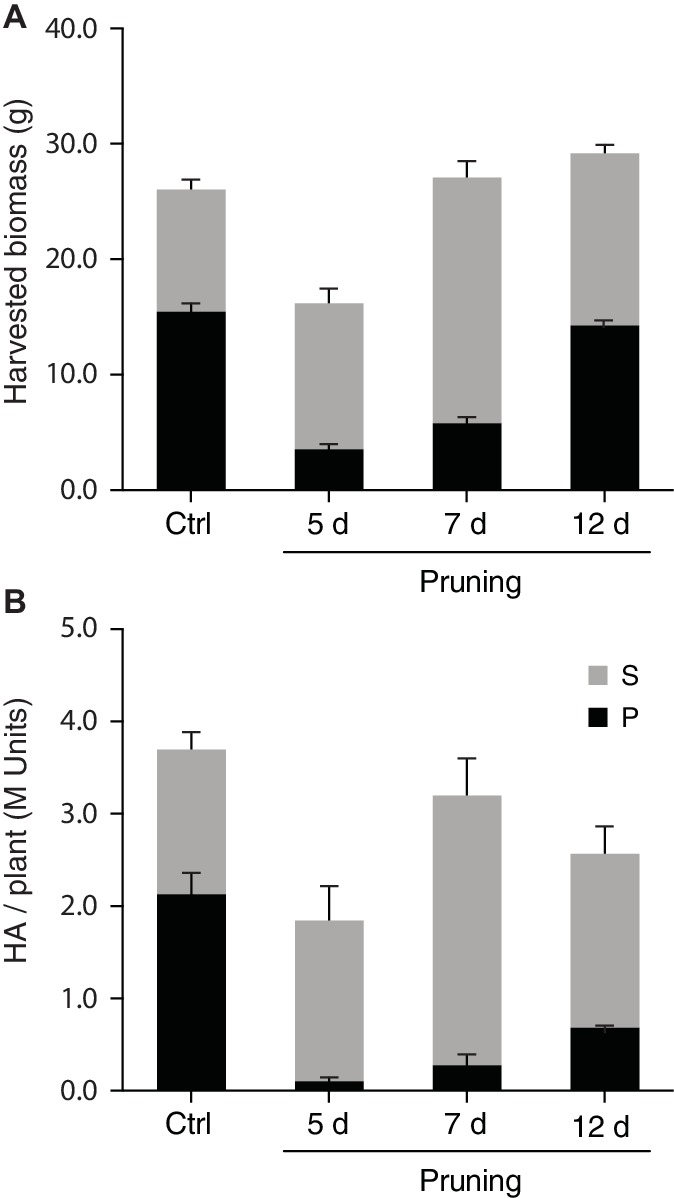
Apex pruning positively influences axillary growth but has no positive impact on H1 yield per plant. Main stem apices were removed 5, 7, or 12 days after seedling transplantation, and the pruned plants then left to grow in greenhouse for an additional 16, 14, or 9 days, respectively, before vacuum infiltration and transfer in growth chamber. Harvested biomass was recorded **(A)**, and total H1 yield determined **(B)**, for P and S leaf production units following heterologous protein expression. Each bar is the mean of four independent (replication) values ± se. Ctrl, control, no-pruning treatment. P, leaf production units P1–P3; S, leaf production units S1–S3.

**Figure 5:**
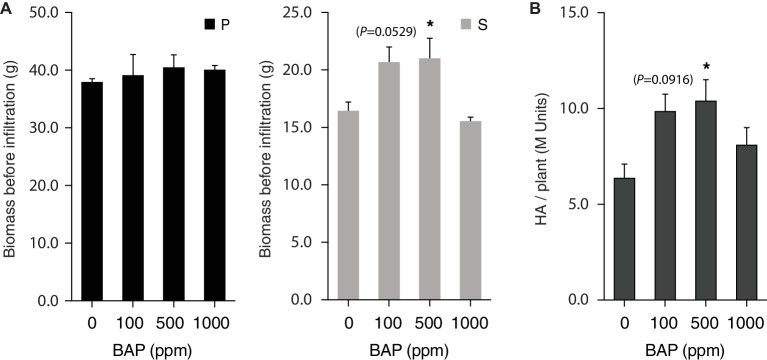
6-BAP cytokinin promotes axillary growth and total H1 yield per plant. Plants treated 7 days (100 and 500 ppm) or 12 days (1,000 ppm) after seedling transplantation and left to grow in greenhouse for an additional 13 or 8 days before vacuum infiltration for H1 expression in growth chamber. Harvested biomass was recorded **(A)**, and total H1 yield per plant determined **(B)**, for P and S leaf production units following heterologous protein expression. Each bar is the mean of three independent (replication) values ± se. Asterisks indicate statistically significant differences compared to the control treatment (post-ANOVA Tukey’s test; *p* < 0.05). P, production units P1–P3; S, production units S1–S3.

Apex pruning and foliar application of synthetic cytokinins are common cultural practices to control plant architecture for improved yield or trait quality in greenhouse settings. These two cultural practices exert a strong downregulating effect on shoot apical dominance over axillary buds, to induce bud outgrowth and axillary stem growth *via* complex signaling pathways dependent on auxins, cytokinins, strigolactones, gibberellins, sugars, and an array of protein receptors and gene regulators (Ferguson and Beveridge, 2009; Domagalska and Leyser, 2011; Rameau et al., 2015). Here, we tested the potential of mechanical pruning and 6-BAP treatment to promote H1 yield in *N. benthamiana via* a positive effect on axillary stem growth (Figures 4, 5). Apex pruning 7 or 12 days after seedling transplantation had little impact on total biomass at harvest but showed strongly divergent effects on growth of the P and S leaf production units, to generate leaf biomass composed of S leaves at 50–80% compared to less than 35% for untreated control plants (Figure 4A). By comparison, 6-BAP treatment had little impact on P leaves but a positive impact on axillary growth, to give a fresh biomass increased by 15% at harvest, mostly explained by a 35–40% increase of S leaf biomass (Figure 5A). Despite positive effects of both treatments on axillary growth, pruning and 6-BAP had strongly divergent impacts on total H1 yield per plant (Figures 4B, 5B). Whereas apex pruning treatments showed null or negative effects on total H1 yield, 6-BAP treatments increased this variable from ~6 M H1 units/plant in untreated plants to more than 10 M units/plant in treated plants, for a relative yield increase of 65–75% in plants given a 6-BAP working dose of 100 or 500 ppm 7 days post-transplantation. Additional studies are now needed to decipher the physiological effects of both cultural practices on H1 yield changes at the plant scale. The negative impact of apex pruning on H1 yield/plant was likely associated to some extent with the removal of young, high-expressing leaves on the main stem, but the large yield reduction observed in P units given the biomass produced suggests additional effects *in planta*. Likewise, the positive impact of 6-BAP on H1 yield/plant was associated with an increased biomass of the S units, but an H1 yield increase of 65–75% measured in plants treated with the synthetic hormone suggests additional causes given the total biomass increase of only 15% on a whole plant basis.

## Conclusion

Cultural conditions have a significant impact on recombinant protein yields in *N. benthamiana* (Fujiuchi et al., 2014, 2017; Matsuda et al., 2017, 2018; Shang et al., 2018). Our goal in this study was to document eventual links between the effects of some current cultural practices on plant development and the production yield of influenza virus hemagglutinin H1, a promising vaccine antigen, in leaves of *N. benthamiana* used as a host for protein expression. Plants grown under different supplemental lighting regimes, submitted to tip pruning, or treated with the axillary growth-promoting hormone 6-BAP were agroinfiltrated to express the recombinant antigen for a week in growth chamber. In accordance with previous accounts on the distribution of recombinant mammalian antibodies in tobacco leaves (Stevens et al., 2000; Buyel and Fischer, 2012), we showed the importance of young leaves on H1 yield per plant, unlike older leaves accounting for a significant part of the plant biomass but contributing little to total antigen titer. We also documented the key contribution of axillary stem leaves, which under certain conditions contributed more than 50% of the antigen yield despite accounting for less than a third of the total biomass. These findings underline the relevance of both considering main stem and axillary stem leaves for a valid monitoring of heterologous protein expression in *N. benthamiana*. They also support the practical potential of 6-BAP in greenhouse settings to modulate host plant architecture for improved protein yields. Additional studies will be welcome in coming years to test the general effectiveness of this approach with other recombinant proteins and to decipher the physiological effects of cytokinins in agroinfiltrated leaves during recombinant protein expression. Studies will also be welcome to further assess the contribution of oldest leaves on the main stem, paying attention in particular to their impact on plant growth before leaf agroinfiltration.

## Data Availability

No datasets were generated or analyzed for this study.

## Author Contributions

M-CG contributed to the conception of the project, to the experimental design, to the experiments, to data acquisition and processing, and to the first draft of the manuscript. LG contributed to the experimental design, to the experiments, to data acquisition, and to the coordination of the project. MG, A-MM, and A-CL contributed to the experimental design and to the experiments. GÉ and NB contributed to the experimental design. MM, M-AD, AG, and SP contributed to the conception of the study and to the experimental design. DM contributed to the conception of the project, to the experimental design, to data processing, and to the writing of the manuscript.

### Conflict of Interest Statement

M-AD, NB, and MM are currently employed by Medicago. Several coauthors on this manuscript are named inventors on a patent describing the effects and uses of growth-promoting hormones to improve recombinant protein yields in plants.

The remaining authors declare that the research was conducted in the absence of any commercial or financial relationships that could be construed as a potential conflict of interest.
