# Supplementary material for: Production of Biopharmaceuticals in Nicotiana benthamiana—Axillary Stem Growth as a Key Determinant of Total Protein Yield
Source: Front Plant Sci. 2019 Jun 11;10:735. doi: 10.3389/fpls.2019.00735 (PMC6579815; doi:10.3389/fpls.2019.00735)
Supplement: Supplementary file 1 [file Table_1.pdf]

SUPPLEMENTARY TABLE S1 | Relative contribution of leaf production units to total biomass and H1 antigen yield per plant [¥]

**Light treatment**<br>. Leaf production unit	**Contribution to yield per plant (%)**	**Contribution to total biomass (%)**	**Yield/biomass ratio**
**Treatment 1.** 16 h | 80 µmol/m²·s			
. P1	21.1	12.6	1.7
. P2	43.4	33.3	1.3
. P3	12.7	29.8	0.4
. S1	4.5	5.3	0.8
. S2	7.6	7.1	1.1
. S3	10.6	11.9	0.9
. P	77.3	75.6	1.0
. S	22.7	24.4	0.9
**Treatment 2.** 16 h | 160 µmol/m²·s			
. P1	25.2	11.3	2.2
. P2	37.0	27.0	1.4
. P3	11.8	34.4	0.3
. S1	6.3	5.3	1.2
. S2	9.5	7.5	1.3
. S3	10.1	14.4	0.7
. P	74.0	72.7	1.0
. S	26.0	27.3	1.0
**Treatment 3.** 24 h | 80 µmol/m²·s			
. P1	14.6	10.1	1.4
. P2	37.4	26.5	1.4
. P3	17.0	34.3	0.5
. S1	7.3	6.6	1.1
. S2	12.0	8.4	1.4
. S3	11.7	14.0	0.8
. P	69.0	71.0	1.0
. S	31.0	29.0	1.1
**Treatment 4.** 24 h | 160 µmol/m²·s			
. P1	18.2	8.5	2.1
. P2	28.2	23.4	1.2
. P3	11.5	30.8	0.4
. S1	13.8	7.2	1.9
. S2	15.9	11.1	1.4
. S3	12.3	19.0	0.6
. P	57.9	62.7	0.9
. S	42.1	37.3	1.1

[¥] Data are expressed relative total leaf biomass or H1 antigen yield per plant (100%). Each value is the mean of 14 independent (replication) values.
